# Genomic insights into *Campylobacter jejuni* from Norwegian broilers: high genetic diversity and limited persistence on farms

**DOI:** 10.1186/s12866-026-04802-5

**Published:** 2026-02-04

**Authors:** Kristin Sæbø Pettersen, Anne Kijewski, Madelaine Norström, Solveig Sølverød Mo

**Affiliations:** Norwegian Veterinary Institute, Elizabeth Stephansens vei 1, Ås, 1433 Ås Norway

**Keywords:** Food safety, Surveillance, Broilers, Campylobacter jejuni, Whole genome sequencing, Zoonotic pathogen, Multilocus sequence typing

## Abstract

**Background:**

Campylobacteriosis is the most common foodborne illness in Norway, and consumption of fresh broiler meat is identified as a significant risk factor for human campylobacteriosis. Up to 2024, data from the Norwegian surveillance programme for *Campylobacter* suggest that a limited number of farms account for the majority of positive flocks. We therefore analysed *Campylobacter* spp. prevalence from 2009 to 2022 and sequenced biobanked isolates collected from flocks on farms with recurrent *Campylobacter* spp. positive flocks (case) and flocks from farms with a few and sporadically *Campylobacter* spp. positive flocks (control) in the period 2011–2022. The aim was to determine if the same *C. jejuni* strain persisted on case farms over time and to compare isolates across farms to investigate whether clonal spread of *C. jejuni* occurs in Norwegian broiler production. Further, we wanted to investigate whether isolates from case farms carry genes that favour infectiousness, colonisation and persistence in broilers or their environment, compared to isolates from control farms.

**Results:**

We identified 69 control farms (75 isolates) and 15 case farms (42 isolates), resulting in cultivation of 117 isolates, all from unique flocks for WGS. Overall, 40 different MLST profiles were identified. Most isolates from case farms had different MLST profiles across years, suggesting introduction of new strains rather than persistent strains across years. ST-45 was the most frequently reported (*n* = 27, 23.1%) ST overall, reported in 9 of 42 (21.4%) isolates from case farms and 18 of 75 (24.0%) isolates from control farms. Further investigation of the isolates with cgMLST, suggested some, but not widespread, clonal dissemination, and that persistence of *Campylobacter* spp. strains on Norwegian farms is rare. The strains from case farms were significantly associated with the three genes *pglD*, *flgG* and *legH*.

**Conclusions:**

This current case control study indicates that *C. jejuni* strains do not persist in *Campylobacter* spp. positive Norwegian broiler farms across years.

**Supplementary Information:**

The online version contains supplementary material available at 10.1186/s12866-026-04802-5.

## Background

*Campylobacter* spp. are the leading cause of food- and waterborne diarrheal disease in the Norwegian population [1]. In 2024, 3091 campylobacteriosis cases were reported to the Norwegian Surveillance System for communicable diseases (MSIS) [2]. Together with non-disinfected water, unpasteurized milk and open land grown food, fresh poultry meat is an important source for human campylobacteriosis [3, 4]. *Campylobacter* infections are usually non-serious and self-limiting, with typical symptoms like diarrhoea and fever, but has the potential to cause severe disease [5]. A subset of patients also develop debilitating/chronic sequela such as Guillain-Barré syndrome (GBS), inflammatory bowel disease (IBD), reactive arthritis (ReA), Reiter’s syndrome or irritable bowel syndrome (IBS) [6]. *Campylobacter jejuni* and *Campylobacter coli*, are the species mostly associated with human disease, with *C. jejuni* being responsible for approximately 90% of the cases in countries such as the United States and United Kingdom [7]. *C. jejuni* have a few distinct properties that distinguish it from other common food borne pathogens, they are for example thermophilic (37–42 °C), strictly microaerophilic (≤ 5% O_2_) and capnophilic (thrives in environments with high CO_2_ concentrations) [8]. Its survival in the environment is drastically influenced by temperature, moisture and the possibility of forming biofilms [9].

Due to high prevalence of *Campylobacter* infections in Norway, and the identification of poultry as a significant source, a national *Campylobacter* surveillance programme [10] was implemented in 2002, targeting commercially produced broilers slaughtered at maximum 50 days of age. The aim of the programme is to reduce *Campylobacter* exposure at consumer level. In 2022, a total of 114 279 tons of poultry meat were produced in Norway (excluding geese, hens and roosters), with broilers covering 92% of the Norwegian market. Of these, only 0.6% of the broilers were organic [11]. Organic and free-range broilers are generally more frequently *Campylobacter*-positive [12] but contributes to only a limited fraction of the total Norwegian production and are therefore not included in the national surveillance programme. Data from the *Campylobacter* spp. surveillance programme has shown that a few farms have recurrent positive flocks, both within and across years. The reason for this is, however, unknown. There are theories as to whether this tendency for recurring *Campylobacter* positive flocks is connected to, or caused by some strains’ abilities to survive and endure better in farm environments, disinfection/hygiene measures [13, 14], climate and seasonal differences [15] or by persistent external transmission sources that contaminates the flocks, (e.g. wild birds, rodents, insects like flies, drinking water, open stored feed supplies, or farm personnel vectors for transmission) [16]. To our knowledge, the potential genetic difference between strains from farms with recurrent *Campylobacter* positive flocks, versus strains from sporadic positive farms, has not been studied in depth.

The aim of this study was to genetically characterize *Campylobacter* spp. isolates from flocks on conventional broiler farms with recurrent positive flocks and compare them with isolates from flocks from farms with sporadic positive flocks. Key objectives were to (i) determine whether the same strain persisted on farms with recurrent positive flocks over several years, (ii) determine the genetic relationship between isolates to clarify whether there is clonal dissemination of *Campylobacter* spp. in Norwegian broiler production, and (iii) to determine whether isolates from recurrent positive farms harbour any genes not detected in isolates from farms with sporadic *Campylobacter* spp. positive flocks, which can facilitate survival, infection, colonization and/or persistence.

## Methods

### Case and control definition

Case- and control farms were selected based on the results from the Norwegian action plan on *Campylobacter* spp. in broiler production from 2009 to 2022 [17].

Farms were defined as cases (recurrent) if *C. jejuni* was detected in at least one flock per year for at least four consecutive years from 2009 to 2022.

Farms were defined as controls (sporadic) if they had (a) maximum two positive flocks per year, (b) positive flocks in only one year during a four-year period, (c) positive flocks in no more than two years in total from 2009 to 2022.

Data from the Norwegian monitoring programme for antimicrobial resistance in food, feed and animals (NORM-VET) and the Norwegian surveillance programme for *Campylobacter* spp. are stored in the Laboratory Information System (LIMS) at the Norwegian Veterinary Institute. Data extraction and management was performed using SAS-PC System^®^ v 9.4 for Windows (SAS Institute Inc. Cary. NC. USA). In brief, data from the two programmes were extracted for the study period and joined by the unique Farm Identification numbers. Only farms and flocks which fulfilled the criteria were selected for the analyses.

### Bacterial isolates

*C. jejuni* isolates from positive flocks (one isolate per flock) have been biobanked at the NVI every second year from 2011 to 2022 in the NORM-VET programme and were available for the present study [18]. Isolates were recovered from the biobank and plated on blood agar (Oxoid, Basingstoke, UK) and incubated for 44 ± 4 h at 41.5 °C in microaerophilic atmosphere generated using Oxoid CampyGen™ (Oxoid, Basingstoke, UK).

### DNA extraction

DNA was extracted manually from fresh colonies using the QIAmp DNA Mini kit (Qiagen, Hilden, Germany) following the manufacturers description. The optional RNAse A step was included, and Tris (10 mM, pH 8) was used as elution buffer. DNA purity and concentration was determined using MySpec (VWR, Radnor, Pennsylvania, USA) and Tecan Spark Fluorometer (Tecan, Männedorf, Switzerland) with the Qubit broad range kit (Invitrogen, Carlsbad, USA) respectively.

### Whole genome sequencing

Library preparation for whole genome sequencing (WGS) was done using the Nextera Rm DNA Flex library preparation kit (Illumina, San Diego, USA). The prepared libraries were sequenced on an Illumina NextSeq 550 resulting in 150 bp paired-end reads (*n* = 63 isolates), or on an Illumina MiSeq resulting in 300 bp paired-end reads (*n* = 68 isolates). All raw sequencing data were uploaded to the European Nucleotide Archive (ENA) with the study accession number: PRJEB102489. Accession numbers for each genome can be found in Supplementary Table S1.

### In silico analysis of sequencing data

The quality of the raw sequencing reads was evaluated using FastQC v. 0.73 [19]. Possible contamination was revealed by Kraken2/Bracken v. 2.11/2.6.1 [20, 21]. Multilocus sequence types (MLST) were determined using the Galaxy-version of MLST [22] and the *Campylobacter* MLST scheme described by Dingle and colleagues [23] and hosted by PubMLST (https://pubmlst.org/) [24]. Genomes were assembled using Shovill v. 1.0.4 [25], and the quality of the assembly assessed using Quast v. 5.0.2 [26].

Core-genome MLST (cgMLST) was performed using chewBBACA v. 3.3.1 [27] on isolates belonging to the same ST to evaluate the genetic relatedness of isolates across farms for STs with more than five isolates reported (STs 21, 45, 48 and 230). This was done using the ALPPACA pipeline [28] and the cgMLST schema described by Cody et al. [29]. Isolates with novel MLST alleles and/or MLST profiles were submitted to PubMLST for assignment of allele- and/or MLST numbers [24]. Isolates with ≤ 14 allele differences were defined as clusters [30].

To determine if certain genes were significantly associated with isolates from case- or control farms, respectively, all genomes were subjected to a core gene analysis using Panaroo v. 1.5.1 [31] in the ALPPACA pipeline [28]. Genes present in all isolates were excluded. To test if significant associations were present between genes and case or control isolates, respectively, we ran Fischer’s exact test in R v. 4.4.3 [32] using the fisher.test with Bonferroni correction to avoid false positive correlations using p.adjust and method = “bonferroni”. A *p*-value < 0.05 was considered significant. Further, a literature search was performed to determine the function of genes significantly associated with case isolates, to determine their biological function and to discuss their potential fitness impact. Phylogenetic trees were visualized in RStudio v. 4.4.3 [32] using the ggtree package v. 3.14.0 [33].

## Results and discussion

**Fig. 1:**
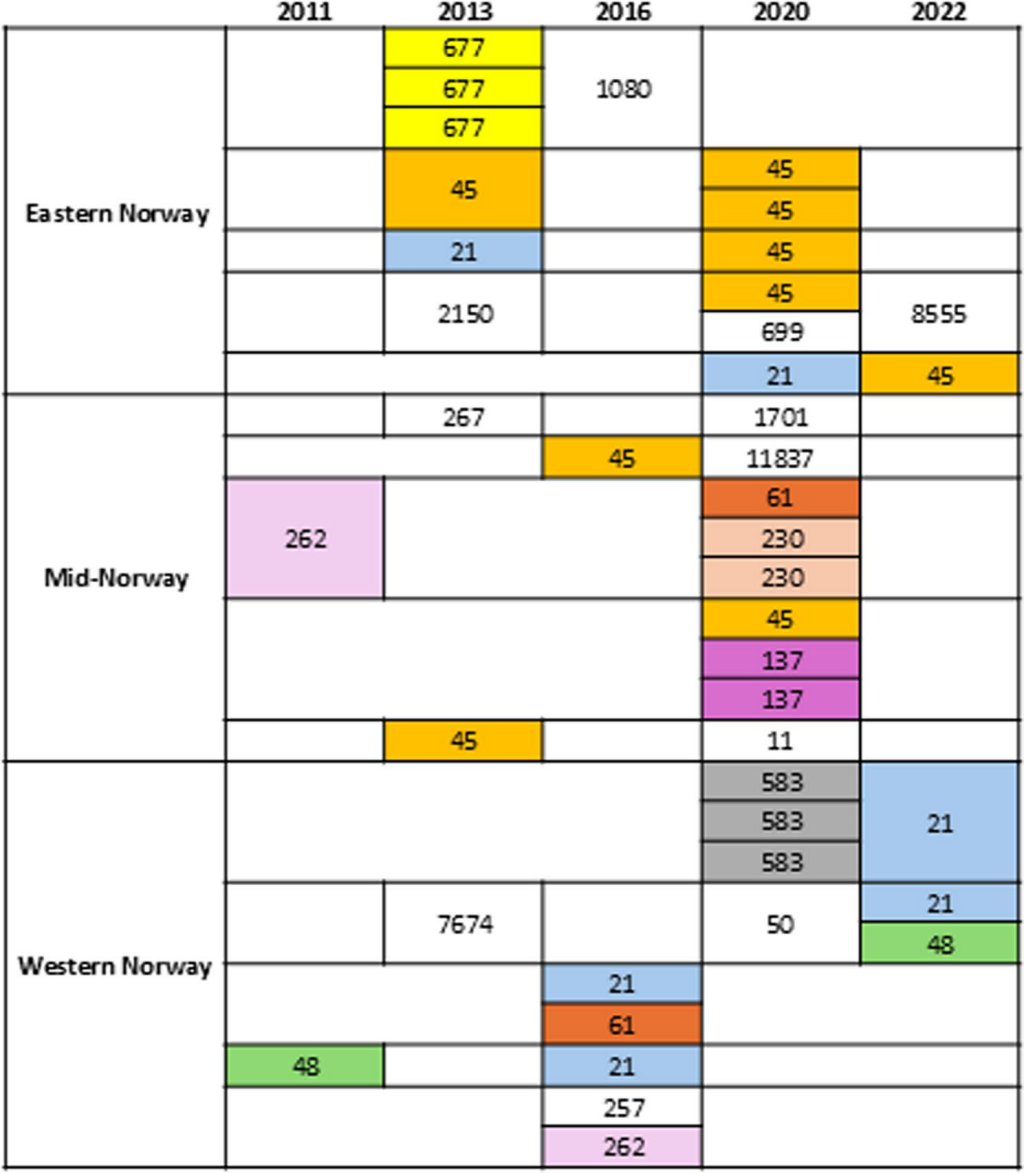
Distribution of *Campylobacter jejuni* isolates collected from case farms and their respective multilocus sequence types by region and year. Sequence types reported in more than one isolate are coloured by ST type. Each case farm is represented by a row indicated in bold lines. Each isolate represents a single flock

Initially, 69 control farms and 28 case farms were included. Due to only a single isolate being available for WGS, thirteen case farms were excluded as analysis of persistence would not be possible. Further, one case farm was excluded due to contamination of one of the *C. jejuni* isolates, leaving only a single isolate from this farm. After exclusion, 42 isolates from 15 case farms and 75 isolates from 69 control farms were included in the study (Supplementary Table S1). Case farms were equally distributed between Eastern-, Mid- and Western-Norway (Fig. 1).

In five case farms, *C. jejuni* with the same ST were detected in different flocks reared the same year. In one of these five case farms, isolates with the same ST were also detected across years (2013 and 2020, respectively) (Fig. 1). In the remaining ten case farms, isolates belonged to different STs, indicating new introduction of *C. jejuni* rather than persistence of one strain.

Overall, 39 different STs were present, 19 of which were only represented by single isolates. In case farms, 21 different STs were observed (Fig. 1), while 32 different STs were detected in control farms (Supplementary Figure S1). Overall, ST-45 was the most frequently reported (*n* = 27), followed by ST-21 (*n* = 10), ST-48 (*n* = 7) and ST-230 (*n* = 7) (Supplementary Table S1). ST-45 was detected in 9 of 42 (21.4%) isolates from case farms and 18 of 75 (24.0%) isolates from control farms. In Europe, ST-45 is one of the five most commonly observed STs, along with ST-21, ST-48, ST-50 and ST-5136 [34]. In Norway, samples are collected for the *Campylobacter* surveillance programme from May 1 st - October 31 st due to the known peak in positive flocks during this time of year [17], possibly due to weather conditions favouring *Campylobacter* survival and growth [35]. Seasonal variations in *Campylobacter* spp. prevalence have also been reported from other countries, such as Finland and the United Kingdom (UK), in contrast to New Zealand where there is less seasonal weather variation [36, 37]. In both Finland and the UK, ST-45 has been abundant and associated with seasonal summer peaks in *Campylobacter* prevalence, while it was less common in New Zealand [37]. The high incidence of ST-45 observed in this study, mainly in Mid- and Eastern-Norway, may therefore be related to geography and/or seasonal weather variations. ST-45 has previously been shown to withstand heat stress and oxidative stress better than other STs and to colonize penguins in Antarctica [38, 39].

### Cluster analysis

**Fig. 2:**
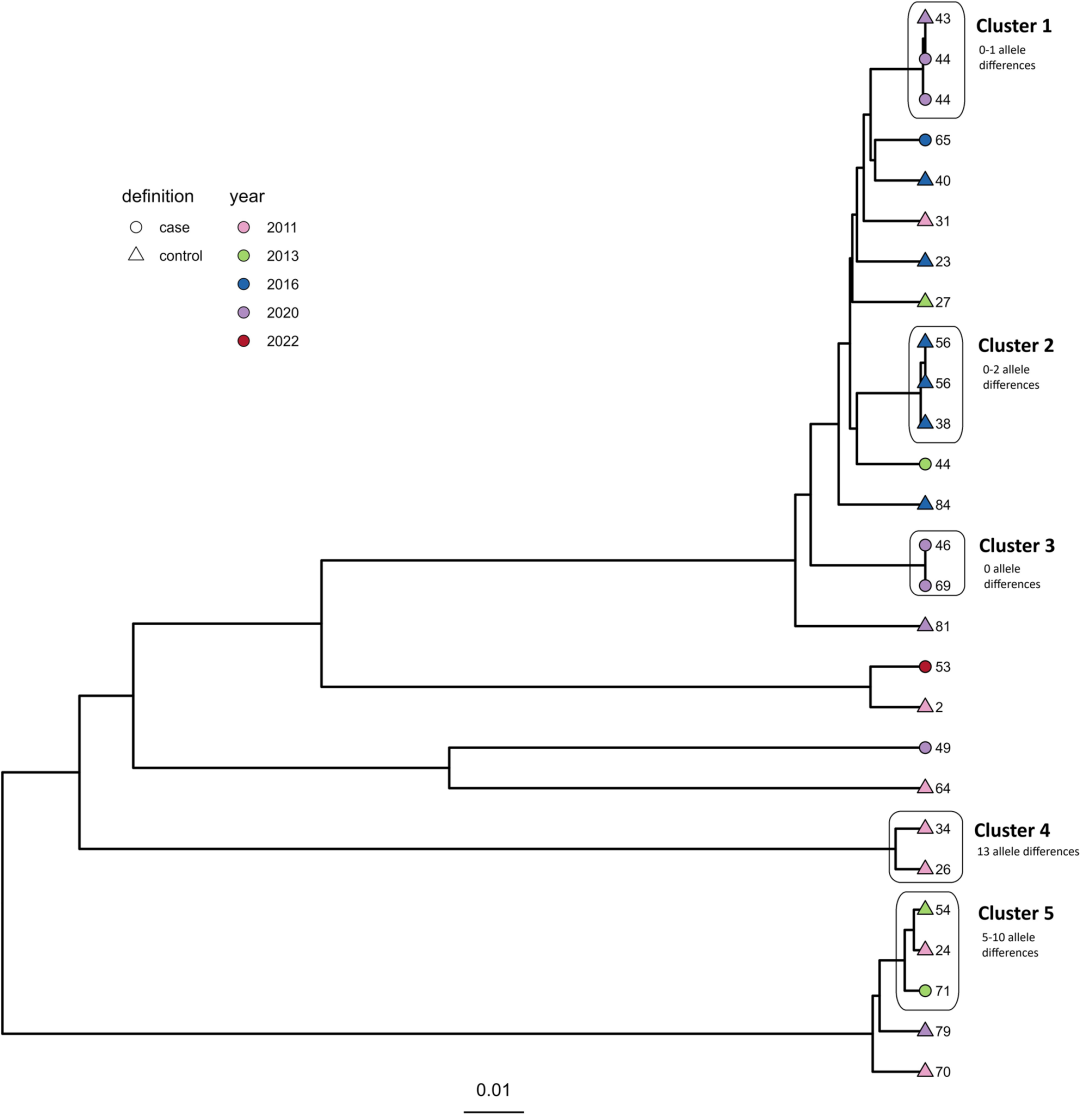
Clustering dendrogram from the cgMLST analysis of the ST-45 isolates (*N* = 27). Shape of tippoints represent case (circle) and control (triangle) farms, while colour of tippoints represents year of isolation

For ST-45, 0–544 allele differences were observed overall between all included isolates. Five clusters were detected (Fig. 2).

Cluster 1 consisted of three isolates from 2020 differing by 0–1 alleles. Two isolates originated from different flocks reared simultaneously on a case farm, while the third was from a control farm. Interestingly, a third isolate from the case farm originating from 2013 did not cluster together with the two isolates from 2020, but differed by 40–41 alleles, indicating new introduction rather than persistence of an ST-45 strain over time. Cluster 2 included three isolates from 2016 originating from three different flocks from two control farms and differed by 0–2 alleles. Two flocks from the same farm were reared simultaneously, but in different houses. Cluster 3 included two isolates with no allele differences. These originated from two different case farms and were isolated in 2020. In Cluster 4, there were two isolates from 2011 originating from two control farms, differing by 13 alleles. Cluster 5 included three isolates with 5–10 allele differences originating from one case (isolated in 2011) and two control farms (isolated in 2013). Overall, the findings indicate possible persistence of ST-45 strains on farms and/or a common ancestor source of *C. jejuni* ST-45 contamination, although new introductions to farms also seem to occur.

**Fig. 3:**
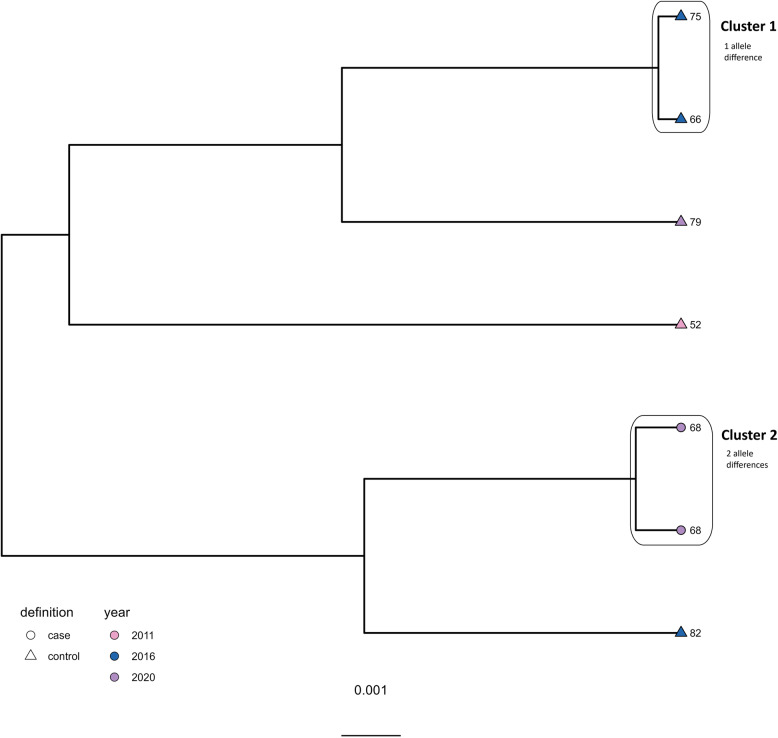
Clustering dendrogram from the cgMLST analysis of the ST-21 isolates (*N* = 10). Shape of tippoints represent case (circle) and control (triangle) farms, while colour of tippoints represents year of isolation

For ST-21 (Fig. 3), a total of 4–478 allele differences were observed overall, and one cluster including five isolates was detected. The isolates in Cluster 1 originated from two different case farms and three different control farms and differed by 4–14 alleles, although isolated between 2011 and 2016. The limited number of allele differences observed indicates a possible common source of *C. jejuni* ST-21 contamination.

**Fig. 4:**
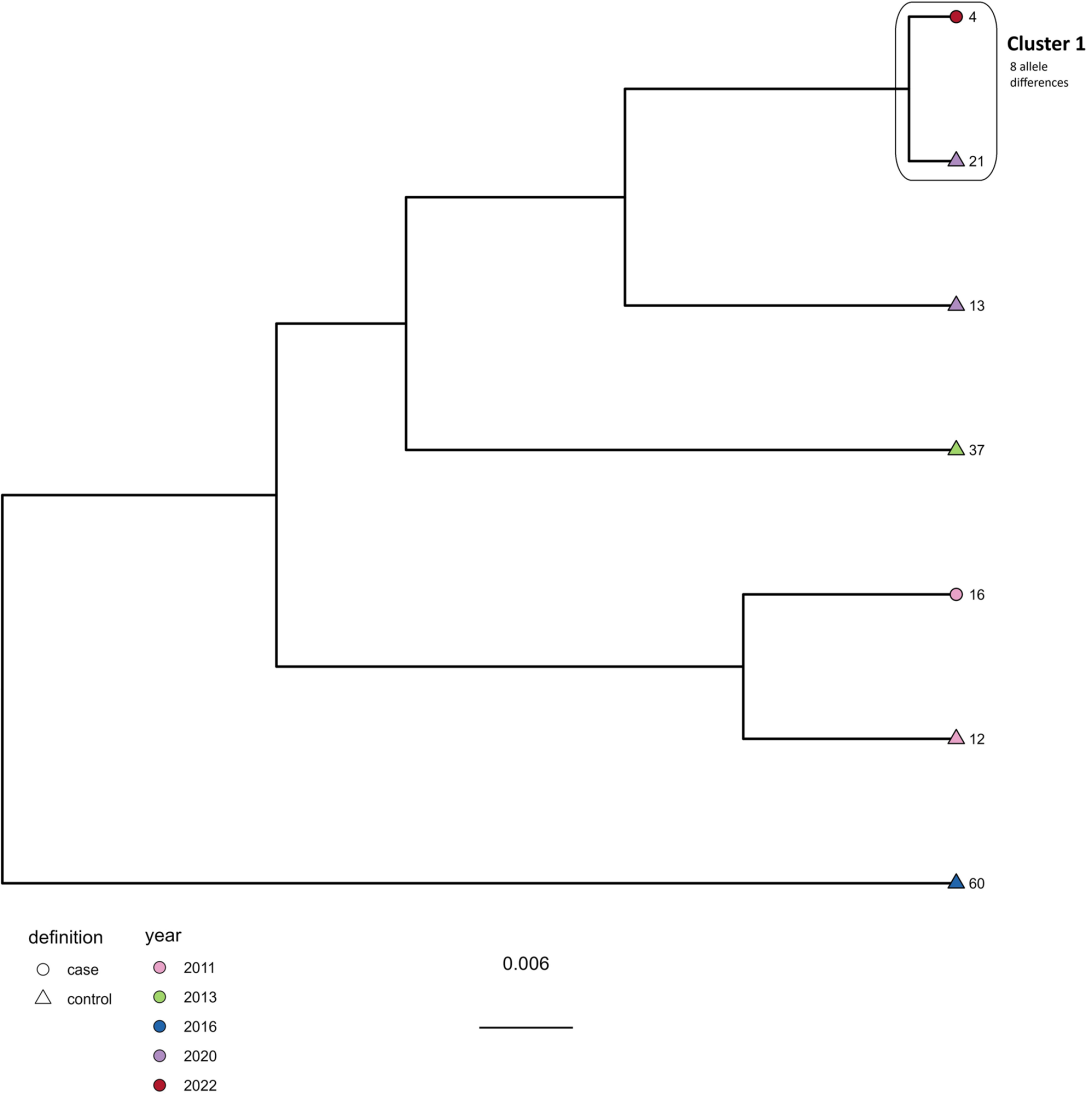
Clustering dendrogram from the cgMLST analysis of the ST-48 isolates (*N* = 7). Shape of tippoints represent case (circle) and control (triangle) farms, while colour of tippoints represents year of isolation

For ST-48 (Fig. 4), one cluster was detected including two isolates from different control farms, one from 2020 and the other from 2022. There were eight allele differences within the cluster, and overall, 8–247 allele differences within the ST. The limited number of allele differences between isolates from different farms and years suggest a common source of *C. jejuni* ST-48 contamination persisting over time.

**Fig. 5:**
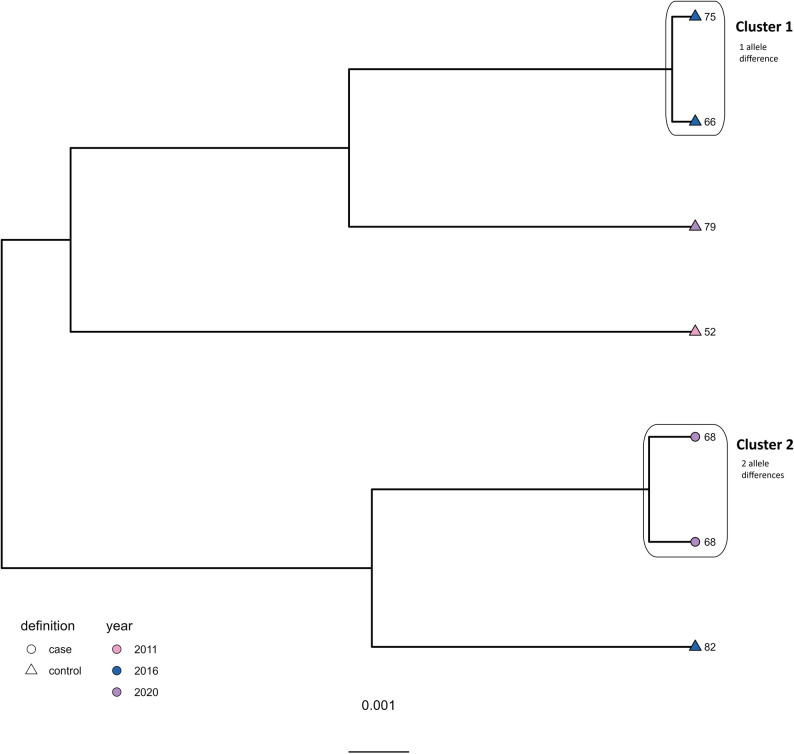
Clustering dendrogram from the cgMLST analysis of the ST-230 isolates (*N* = 7). Shape of tippoints represent case (circle) and control (triangle) farms, while colour of tippoints represents year of isolation

For ST-230 (Fig. 5), 1–43 allele differences were identified in total. Cluster 1 included two isolates from 2016 differing by 1 allele originating from two different control farms.

Cluster 2 included two isolates from two different flocks reared at the same case farm in 2020, differing by 2 alleles The findings support possible persistence of *C. jejuni* ST-230 on farms and/or a common source of contamination resulting in detection of closely related ST-230 isolates at different farms. Further, one isolate from 2016 originating from a control farm differed by 14 alleles to one of the isolates in Cluster 2, but with 16 alleles to the other, and was therefore not considered as part of the cluster. However, it is possible that there has been a common source for the strains at these two farms, as isolates differing by ≤ 14 alleles are considered related [30].

We found less diversity among ST-230 isolates compared to ST-21, ST-45 and ST-48. ST-21, ST-45 and ST-48 are all considered to belong to clonal complexes categorized as host generalists [40]. The ST-230 is categorized in CC-45 together with ST-45 [24] and the relatively low genetic diversity observed within ST-230 compared to ST-45 may be due to the relatively lower number of ST-230 isolates observed in the current study. As we only have a limited number of isolates per ST in this study, it is not possible to determine expected genetic diversity per ST. We suggest future studies focusing on how STs evolve in response to different environmental impacts or while persisting in a farm between production cycles. This would increase our understanding of expected number of allele differences between closely related isolates and improve evaluation of *Campylobacter* spp. persistence and dissemination.

Overall, the results from the cgMLST analyses indicate possible circulation and persistence of highly similar *C. jejuni* ST-45, ST-21, ST-48 and ST-230 strains in the broiler production (within and between farms), possibly originating from a common source (i.e. breeder and/or hatchery facility and/or farm environment/other environmental or wildlife source).

The results from the cgMLST analyses underlines that even within the core genome of *C. jejuni*, including 1343 genes [29], there is abundant diversity within each ST. Within ST-45, which had the highest number of allele differences (*n* = 544), a substantial part of the core genome is variable within the ST. This raises the question of how similar the isolates from the same ST’s are phenotypically.

Overall, our results indicate that prolonged persistence of *C. jejuni* on broiler farms in Norway is rare. For 66.7% (10/15) of the case farms, isolates from flocks sampled across years belonged to different STs, indicating new introduction rather than persistence. In five (33.3%) case farms, several *C. jejuni* isolates with the same ST were detected. However, in four of these farms, all isolates with the same ST were isolated within the same year, also indicating introduction of new strains across years. This underlines the importance of general biosecurity measures preventing introduction of pathogens at farm level. However, only a single *C. jejuni* isolate was characterized per sample, and the possibility of multiple different strains co-occurring simultaneously in a flock cannot be excluded, as has been described previously [41]. Further studies including characterization of multiple *C. jejuni* isolates per flock and sample is therefore required to evaluate whether this is common.

### Genes significantly associated with strains from case farms

We identified three genes significantly associated with isolates from case farms, namely *pglD*, *legH* and *flgG*. The pgl cluster including *pglD* encodes the N-glycosylation system in *C.jejuni* which modifies proteins and is considered highly conserved. Both N-and O-linked glycosylation is stated as fundamental requirements for virulence. The *pgl* N-glycosylation in *C. jejuni* is likely to be important for its fitness in environmental, avian and human niches [42]. The gene *legH* encodes for N-acetyltransferase involved in biosynthesis of legionaminic acid, which is used for O-linked glycosylation of flagella proteins, and lack of the *legH* gene may have a negative impact on motility. The *flgG* gene is considered critical for the flagellar assembly [43], and strains lacking this gene is considered to be less motile. Motility is likely important when *C. jejuni* are introduced to new niches such as poultry houses and poultry hosts.

Genes significantly associated with control isolates are presented in Supplementary Table S2.

## Conclusions

The results from this study suggest that long-term persistence of *C. jejuni* on Norwegian broiler farms is rare. Only one out of 15 case farms had the same *C. jejuni* ST isolated from flocks across different years, and the relatively high number of allele differences between these isolates indicated new introductions rather than persistence at the farms. cgMLST analyses also revealed some clustering of isolates across farms, suggesting possible clonal dissemination, possibly from a common source. However, most clusters were small and did not indicate widespread clonal dissemination. The study supports previous literature, which has suggested that seasonality and geography may have an influence on the prevalence of certain STs, especially ST-45. While the study did not find persistence of *C. jejuni* strains to be common on Norwegian farms, we found three genes significantly associated with strains from case farms, all associated with motility and/or increased fitness While the carriage of these genes suggests an advantage for these case strains, more research on a protein level of infection is needed to determine the full impact the carriage of these genes can have on *C. jejuni* infectivity, survival and virulence.

## Supplementary Information

Supplementary Material 1.

Supplementary Material 2.

## Data Availability

Raw reads are available from the European Nucleotide Archive (ENA), study accession number PRJEB102489. Additional data are presented in the supplementary tables.

## Abbreviations

AMR: Antimicrobial resistance
CC: Clonal Complex
cgMLST: Core Genome Multi Locus Sequence Typing
CPS: Capsular Polysaccharide
MLST: Multi Locus Sequence Typing
MSIS: Norwegian Surveillance System for Communicable diseases
NFSA: The Norwegian Food Safety Authorities
QC: Quality Control
ST: Sequence type
WGS: Whole Genome sequencing
