# Supplementary material for: Genomic insights into Campylobacter jejuni from Norwegian broilers: high genetic diversity and limited persistence on farms
Source: BMC Microbiol. 2026 Feb 4;26:244. doi: 10.1186/s12866-026-04802-5 (PMC12990561; doi:10.1186/s12866-026-04802-5)
Supplement: Supplementary file 2 — Supplementary Material 2. [file 12866_2026_4802_MOESM2_ESM.pdf]

	2011	2013	2016	2020
Eastern Norway	45			
		61		
	45			
		45		
		3863		
		2219		
			14455	
	45			
		1367		
		53		
	45			
		637		
		1701		
		48		
			45	
		794		
			45	
		3971		
		13547		
				45
				1080
		945		
				267
			42	
				11
	230			
		45		
		11		
			45	
			45	
Mid-Norway		945		
			45	
		1276		
			61	
			48	
			696	
			21	
				11
	45			

			230	
	3971			
	45			
			3971	
		137		
	21			
			230	
			42	
		3971		
			137	
				45
				230
			3971	
				45
			230	
			5359	
			5359	
			45	
			4080	
**Southern Norway**	677			
**Western Norway**	45			
			19	
	273			
			177	
			177	
	21			
			257	
				257
	48			
				48
		177		
				21
			262	
	334			
			262	
				48

Supplementary Figure S1. Distribution of *Campylobacter jejuni* isolates collected from control farms and their respective multilocus sequence types by region and year. Sequence types reported in more than one isolate are coloured by ST type. Each case farm is represented by a row indicated in bold lines. Each isolate represent a single flock.
